# A pioneering study indicate role of GABRQ rs3810651 in ASD severity of Indo-Caucasoid female probands

**DOI:** 10.1038/s41598-021-86496-5

**Published:** 2021-03-26

**Authors:** Sharmistha Saha, Mahasweta Chatterjee, Swagata Sinha, Kanchan Mukhopadhyay

**Affiliations:** Manovikas Biomedical Research and Diagnostic Centre, Manovikas Kendra, 482 Madudah, Plot I-24, Sector J, EM Bypass, Kolkata, West Bengal 700107 India

**Keywords:** Developmental biology, Genetics, Neuroscience

## Abstract

Alteration in gamma aminobutyric acid (GABA), the principal inhibitory neurotransmitter, is speculated to be a potential risk factor for Autism Spectrum Disorder (ASD) due to an altered expression in the brain. Sensory, social, and emotional deficits of subjects with ASD were reported to be caused by an imbalance between excitatory and inhibitory neurotransmission as well as GABAergic dysfunction caused by inadequate receptor function. We for the first time studied association between ASD and a missense coding variant rs3810651 (I478F) in the GABRQ gene, encoding for one of the subunits of GABA_A_ receptors. Stratified analysis on families with ASD probands (N = 251) and ethnically matched control subjects (N = 250) revealed marginally higher frequency of “A” allele and “AA” genotype in female ASD probands as compared to gender matched controls. Female probands demonstrated higher severity for Verbal communication (χ^2^ = 5.75, *P* = 0.01), Activity level (χ^2^ = 7.26, *P * = 0.007), as well as Level and consistency of intellectual response (χ^2^ = 7.83 *P* = 0.005) in presence of “A/AA” warranting further in-depth investigation on the role of rs3810651 in ASD.

## Introduction

Autism spectrum disorder (ASD) is a highly heritable complex disorder showing steep increase in prevalence throughout the world, including India. ASD is characterized by early childhood symptoms of (1) persistent deficits in social communication & interaction and (ii) restricted repetitive patterns of behaviors and interests^1^, the extent of which vary from mild, moderate to severe. Number of boys affected with ASD is higher as compared to girls^1^. Investigations have shown that genetic, epigenetic, and environmental factors influence ASD symptom severity^2,3^. However, till date, diagnosis is exclusively based on medical history and presenting symptoms^1^. Identification of novel genetic markers which can be used for recognizing ASD and its associated symptom severity thus becomes vital.

Behavioral abnormalities of subjects with ASD are hypothesized to be due to an imbalance in the levels of excitatory/inhibitory neurotransmitters^4^. Gamma aminobutyric acid (GABA), the principal inhibitory neurotransmitter, is crucial for maintaining neuronal plasticity as well as synchronized brain activity through regulation of other neurotransmitters. Accordingly, modulation of GABAergic signaling is now sought as an alternative approach for providing symptomatic remediation in subjects with ASD^5^. GABA_A_ receptor mediates fastest inhibitory synaptic transmission in the central nervous system. Sixteen different subunits determine the receptor's agonist affinity, chance of opening, conductance, and other properties^6^. Amongst these, significant expression of GABA_A_ receptor theta subunit in the brain and a combination of specific subunits was found to reduce affinity of the receptor to GABA^7^. GABA_A_ receptor theta subunit is encoded by the GABRQ gene located at chromosome Xq28 and a truncating rare variant, c.306 G- > C, was detected in one female ASD patient^8^. Another GABRQ coding variant, rs3810651, is a missense polymorphism in the last exon of GABRQ and was explored for association with neurological disorders like migraine, essential tremor and restless leg syndrome^9–12^. However, to the best of our knowledge, till date this variant was not reported to have association with ASD.

We for the first time investigated association between rs3810651 and ASD to find out whether this SNP has any contribution in the etiology of the disorder. Further, due to the reported higher occurrence of ASD in boys and localization of GABRQ in the X chromosome, we have performed gender-based stratified analysis.

## Results

### Comparative case–control genetic association analysis

Genotype of female participants followed the Hardy–Weinberg equilibrium. Population-based analysis failed to show any significant difference in frequency of rs3810651 alleles in all ASD probands, even after stratification based on gender (data not included for brevity). However, stratified analysis based on gender as well as disease severity exhibited marginally higher frequency of the “A” allele and “AA” genotype in the female probands with moderate severity as compared to gender matched controls (Tables 1 and 2; χ^2^ = 4.56 & 6.95 respectively, *P* = 0.03).

**Table 1:** Population based comparative analysis on rs3810651 allelic frequency.

Allele	Control (N = 250)	ASD probands				Male participants					Female participants				
						Control (N = 147)	ASD probands				Control (N = 103)	ASD probands			
		Moderate symptoms (N = 167)	χ^2^ (P)	Severe symptoms (N = 84)	χ^2^ (P)		Moderate symptoms (N = 149)	χ^2^ (P)	Severe symptoms (N = 58)	χ^2^ (P)		Moderate symptoms (N = 18)	χ^2^ (P)	Severe symptoms (N = 26)	χ^2^ (P)
A	0.43	0.46	0.33 (0.56)	0.46	0.17 (0.68)	0.45	0.42	0.27 (0.60)	0.41	0.18 (0.67)	0.41	0.69	**4.56 (0.03)**	0.56	1.36 (0.24)
T	0.57	0.54		0.54		0.55	0.58		0.59		0.59	0.31		0.44	

N.B: Statistically significant difference is presented in bold.

**Table 2:** Population based analysis on rs3810651 genotype frequency of female subjects.

Allele/ genotype	Control	Probands	χ^2^ (P)	Probands with mild/ moderate symptoms	χ^2^ (P)	Probands with severe symptoms	χ^2^ (P)
AA	0.17	0.24	0.98 (0.61)	0.38	6.95 (**0.03**)	0.45	3.75 (0.15)
AT	0.48	0.39		0.62		0.22	
TT	0.35	0.37		0		0.33	

N.B: Statistically significant difference is presented in bold.

### Transmission disequilibrium test (TDT) analysis

Family based analysis revealed a trend for higher maternal transmission of the “A” allele to female probands, though the result was statistically insignificant (Table 3; χ^2^ = 2.16, *P* = 0.14).

**Table 3:** Parental allelic transmission to female probands identified by TDT.

Parents	Allele	T	NT	χ2 (P)
Both	A	0.57	0.50	0.14 (0.70)
	T	0.43	0.50	
Father	A	0.62	0.92	0.54 (0.46)
	T	0.38	0.08	
Mother	A	0.62	0.36	2.16 (0.14)
	T	0.38	0.64	

### Genotype–phenotype association analysis

ASD subjects exhibit varying level of behavioral characteristics which are deviant from neurotypicals. For assessing disease severity, we have used the Childhood Autism Rating Scale 2-Standard Test (CARS2- ST) covering 15 different phenotypes. Values for each phenotype assessed under CARS2-ST varies between 1 and 4 with 0.5 interval and subjects were categorized into mild to moderate (score 30.0–36.5) and severe (score 37.0–60.0) depending on the total score. Female ASD probands showed statistically significant association of the “A” allele and ‘AA’ genotype with cumulative CARS score as is evident from positive Addvalue (Table 4, *P* < 0.03; ST 1). Independent associations were also noticed in the female probands for all the 15 phenotypic domains included under CARS (Table 4, *P* ≤ 0.05). On the other hand, no such statistically significant allelic association was observed for the male probands (Table 4; *P* > 0.05). Female probands stratified based on disease severity demonstrated association of “A/AA” with higher severity for Verbal communication (χ^2^ = 5.75, *P* = 0.01), Activity level (χ^2^ = 7.26, *P* = 0.007), as well as Level and consistency of intellectual response (χ^2^ = 7.83 *P* = 0.005).

**Table 4:** Association between rs3810651 alleles/genotypes and traits of ASD probands (N.B. Only statistically significant values are presented for female probands).

CARS Domain	Allele/Genotype	Male proband (N = 207)		Female proband (N = 44)	
		AddVal	χ^2^(P)	AddVal	χ^2^(P)
Cumulative score	A	+ 0.01	1.09(0.29)	+ 0.04	**4.64(0.03)**
	AA	–	–	+ 0.20	**6.27(0.01)**
Relating to people	A	+ 0.06	0.11(0.74)	+ 0.51	**4.42(0.03)**
	AA	–	–	+ 1.55	**5.37(0.02)**
Imitation	A	+ 0.23	1.48(0.22)	+ 0.46	**3.94(0.05)**
	AA	–	–	+ 1.43	**6.00(0.006)**
Emotional response	A	+ 0.32	2.21(0.14)	+ 0.53	**4.85(0.03)**
	AA	–	–	+ 1.98	**10.02(0.0001)**
Body use	A	+ 0.16	0.91(0.34)	+ 0.43	**4.03(0.04)**
	AA	–	–	+ 1.74	**5.15(0.02)**
Object use	AA	–	–	+ 1.10	**4.42(0.04)**
Adaption to change	A	+ 0.22	1.08(0.30)	+ 0.51	**3.97(0.05)**
	AA	–	–	+ 1.52	**6.13(0.01)**
Visual response	A	+ 0.12	0.36(0.55)	+ 0.45	**3.69(0.05)**
	AA	–	–	+ 1.92	**8.99(0.002)**
Listening response	AA	–	–	+ 1.26	**6.01(0.01)**
Taste, smell and touch	A	+ 0.22	0.91(0.34)	+ 0.48	**3.75(0.05)**
	AA	–	–	+ 1.24	**6.20(0.008)**
Fear or Nervousness	A	+ 0.31	1.25(0.26)	+ 0.77	**5.77(0.02)**
	AA	–	–	+ 2.14	**8.27(0.004)**
Verbal communication	A	+ 0.15	0.74(0.39)	+ 0.48	**4.71(0.03)**
	AA	–	–	+ 1.64	**8.56(0.003)**
Nonverbal communication	A	+ 0.17	0.74(0.39)	+ 0.45	**4.08(0.04)**
	AA	–	–	+ 1.35	**7.25(0.007)**
Activity level	A	+ 0.16	0.84(0.36)	+ 0.71	**6.74(0.009)**
	AA	–	–	+ 1.49	**8.32(0.003)**
Level and consistency of Intellectual Response	A	+ 0.29	2.52(0.11)	+ 0.52	**5.12(0.02)**
	AA	–	–	+ 1.67	**7.43(0.006)**
General impression	A	+ 0.19	1.16(0.28)	+ 0.42	**3.74(0.05)**
	AA	–	–	+ 1.47	**6.41(0.01)**

## Discussion

In silico analysis on X-linked synaptic genes predicted GABRQ A > T: Ile478Phe (i.e. rs3810651) to be non-pathogenic^13^. Genomic DNA samples of ASD patients showed truncating mutations in GABRQ and GABRA3 in two different families with ASD probands^8^ though no association between rs3810651 and ASD was reported^8,13^. On the other hand, rs3810651 was found to be a potential candidate for a common neurological disorder migraine and carriers of rs3810651 “AT” genotype showed increased risk of migraine, which was significant for female subjects^9^. Frequency of the “A” allele was appreciably higher in the subgroup of female migraine patients with ≤ 15 years age of onset and subjects with “AA” genotype showed a lower mean age of onset of migraine attacks^10^. rs3810651 “T” allele exhibited association with improvement in essential tremor^11^. In Major depressive disorder patients, rs3810651 “A” allele showed significant positive association with antidepressant drug response^14^. No significant association of the studied variant was noticed with restless leg syndrome^12^.

Genotype of female participants recruited for the present study followed the Hardy–Weinberg equilibrium. Assumption taken into consideration while calculating Hardy–Weinberg equilibrium was (1) non-consanguineous marriage of parents, (2) same ethnicity, and (3) no genetic drift.

Our investigation for the first time revealed association of this variant with phenotypes of female ASD probands indicating a role of *GABRQ* rs3810651 ‘A’ allele in the severity of ASD associated traits of female probands. In spite of having reasonable number of male probands, we failed to notice any significant association of *GABRQ* rs3810651 in this subgroup. An earlier study on a large dataset showed that female ASD probands with less than 70 IQ had overall more impairments than males with ASD in terms of social-communicative abilities, cognitive and adaptive abilities, and externalizing behaviors^15^. Girls diagnosed with ASD were also reported to have a higher mean total problem score (hyperactivity, anxiety, and conduct, peer, and prosocial problems) and higher frequency of low IQ than boys with ASD^16^. Comparative analysis on neurobehavioral profile exhibited poorer inhibition in females with ASD as compared to males^17^. Females with ASD showed significantly increased grey matter volume in the right anterior cingulate cortex and right cerebellum^18^, brain regions with neuronal activity typically mediated by GABA^19^.

We infer from the present investigation that *GABRQ* rs3810651 may have a specific role in ASD severity which is dependent on gender. Our study has three major drawbacks; (1) limitation in number of female ASD probands. However, it has already been noticed that from human studies to model systems, limited availability of female ASD samples and limited attention to gender as a biological variable have hindered our ability to describe as well as to understand the importance of sex difference in ASD pathology^20^. Hence, further extensive research including larger female ASD samples is warranted to identify the importance of GABRQ gene in the etiology of ASD in sex balanced sample sets. (2) absence of high functioning (HF) ASD patients, i.e. with IQ > 80, in our study group. Our subjects were homogenous for age (i.e. 5.96 years ± 3.31), low IQ (< 80) and limited verbal ability. This could be one of the reasons for our observed higher trait scores in female probands, as low IQ have already been reported to be associated with poorer traits in female ASD patients^15,16^. Having a comparative analysis on subjects with broader ASD phenotypes characterized by a more diverse sample in terms of high functioning vs. low-functioning would help to identify the precise association between GABRQ rs3810651 and ASD phenotype. (3) assessment of traits using CARS2-ST. Analysis of association between GABRQ rs3810651 and ASD using diagnostic criteria mentioned in the DSM-5 and scores obtained through other ASD assessment tools like Autism Diagnostic Observation Schedule along with the Autism Diagnostic Interview-Revised may aid in strengthening our findings. Under the current scenario, we conclude that to identify the importance of GABRQ gene in the etiology of ASD further extensive research is warranted.

## Materials and methods

### Subject selection and sample collection

Indo-Caucasoid nuclear families with ASD probands (N = 251; 207 male & 44 female) and controls (N = 250; 147 male & 103 female) were recruited by child psychiatrists and clinical psychologists on the basis of DSM-IV and/or DSM-5^1,21^ after obtaining informed written consent from participants and/or their legal guardians. Mean age of the probands was 5.96 years ± 3.31 (SD). No high functioning (HF) ASD patient, i.e. with IQ > 80, was available for our study. Hence, recruited ASD subjects were assessed by the Childhood Autism Rating Scale 2-Standard Test (CARS2- ST)^22^ to measure symptom severity and subjects were categorized into mild-moderate and severe groups based on the cumulative CARS score with range of 30–36.5 and 37–60. The study protocol was approved by the Human Ethical Committee of Manovikas Kendra consisting of Scientists, Clinicians, Social worker, Advocate and Administrator (PR-006–14) which follow guidelines of the Indian Council of Medical Research. All the research was performed in accordance with relevant guidelines/regulations of the Committee. Genomic DNA was purified from peripheral blood of study participants following standard protocol^23^.

### Genotyping and statistical analysis

Genotyping of coding region variant, rs3810651 of GABRQ was done by TaqMan-based allelic discrimination assay (Assay ID C__27492635_10, Thermo Fisher Scientific, Singapore). Population- and family-based analyses were performed using UNPHASED v 3.1.7^24^. Quantitative Trait (QT) analysis, to identify association between rs3810651 and phenotypic domains incorporated under the CARS as well as cumulative CARS score, was also performed using UNPHASED (v.3.1.7) program^24^.

## Supplementary Information

Supplementary Information
