# Supplementary material for: A pioneering study indicate role of GABRQ rs3810651 in ASD severity of Indo-Caucasoid female probands
Source: Sci Rep. 2021 Mar 26;11:7010. doi: 10.1038/s41598-021-86496-5 (PMC7997895; doi:10.1038/s41598-021-86496-5)
Supplement: Supplementary file 1 — Supplementary Information [file 41598_2021_86496_MOESM1_ESM.docx]

**A pioneering study indicate role of GABRQ rs3810651 in ASD severity of Indo-Caucasoid female probands**

Sharmistha Saha^a^, Mahasweta Chatterjee^a^, Swagata Sinha^a^ and Kanchan Mukhopadhyay^a*^

^a^Manovikas Biomedical Research and Diagnostic Centre, Manovikas Kendra, 482 Madudah, Plot I-24, Sector J, EM Bypass, Kolkata, West Bengal 700107, India.

*Corresponding author:

Kanchan Mukhopadhyay,

Manovikas Biomedical Research and Diagnostic Centre,

482, Madudah, Plot I-24, Sec.-J, E.M. Bypass, Kolkata - 700107, India.

Tel: 91-033-4001-9179; Fax: 91-033-2442-8275

Email: [drkanchnam@gmail.com](mailto:drkanchnam@gmail.com); [kanchanmvk@yahoo.com](mailto:kanchanmvk@yahoo.com)

**ST 1. Association between rs3810651 alleles/genotypes and traits of ASD probands.**

| **CARS Domain** | **rs3810651**  **Allele/Genotype** | **Male proband (n=207)** | | **Female proband (n=44)** | |
| --- | --- | --- | --- | --- | --- |
|  |  | **AddVal** | **χ^2^(P)** | **AddVal** | **χ^2^(P)** |
| Cumulative CARS score | A | +0.01 | 1.09(0.29) | +0.04 | **4.64(0.03)** |
|  | T | -0.01 |  | -0.04 |  |
|  | AA | - | - | +0.20 | **6.27(0.01)** |
|  | AT | - | - | -0.20 | 2.21(0.14) |
|  | TT | - | - | -0.20 | 0.80(0.37) |
| Relating to people | A | +0.06 | 0.11(0.74) | +0.51 | **4.42(0.03)** |
|  | T | -0.06 |  | -0.51 |  |
|  | AA | - | - | +1.55 | **5.37(0.02)** |
|  | AT | - | - | -1.53 | 1.49(0.22) |
|  | TT | - | - | -1.61 | 1.01(0.31) |
| Imitation | A | +0.23 | 1.48(0.22) | +0.46 | **3.94(0.05)** |
|  | T | -0.23 |  | -0.46 |  |
|  | AA | - | - | +1.43 | **6.00(0.006)** |
|  | AT | - | - | -1.46 | 2.64(0.10) |
|  | TT | - | - | -1.38 | 0.45(0.50) |
| Emotional response | A | +0.32 | 2.21(0.14) | +0.53 | **4.85(0.03)** |
|  | T | -0.32 |  | -0.53 |  |
|  | AA | - | - | +1.98 | **10.02(0.0001)** |
|  | AT | - | - | -2.02 | 3.47(0.06) |
|  | TT | - | - | -1.89 | 0.50(0.48) |
| Body use | A | +0.16 | 0.91(0.34) | +0.43 | **4.03(0.04)** |
|  | T | -0.16 |  | -0.43 |  |
|  | AA | - | - | +1.74 | **5.15(0.02)** |
|  | AT | - | - | -1.72 | 1.59(0.21) |
|  | TT | - | - | -1.77 | 0.83(0.36) |
| Object use | AA | - | - | +1.10 | **4.42(0.04)** |
|  | AT | - | - | -1.10 | 1.46(0.22) |
|  | TT | - | - | -1.12 | 0.63(0.43) |
| Adaption to change | A | +0.22 | 1.08(0.30) | +0.51 | **3.97(0.05)** |
|  | T | -0.22 |  | -0.51 |  |
|  | AA | - | - | +1.52 | **6.13(0.01)** |
|  | AT | - | - | -1.55 | 2.75(0.10) |
|  | TT | - | - | -1.45 | 0.44(0.51) |
| Visual response | A | +0.12 | 0.36(0.55) | +0.45 | **3.69(0.05)** |
|  | T | -0.12 |  | -0.45 |  |
|  | AA | - | - | +1.92 | **8.99(0.002)** |
|  | AT | - | - | -1.99 | 3.67(0.06) |
|  | TT | - | - | -1.79 | 0.21(0.65) |
| Listening response | AA | - | - | +1.26 | **6.01(0.01)** |
|  | AT | - | - | -1.26 | 1.72(0.19) |
|  | TT | - | - | -1.26 | 0.59(0.44) |
| Taste, smell and touch | A | +0.22 | 0.91(0.34) | +0.48 | **3.75(0.05)** |
|  | T | -0.22 |  | -0.48 |  |
|  | AA | - | - | +1.24 | **6.20(0.008)** |
|  | AT | - | - | -1.31 | 3.15(0.08) |
|  | TT | - | - | -1.13 | 0.30(0.59) |
| Fear or Nervousness | A | +0.31 | 1.25(0.26) | +0.77 | **5.77(0.02)** |
|  | T | -0.31 |  | -0.77 |  |
|  | AA | - | - | +2.14 | **8.27(0.004)** |
|  | AT | - | - | -2.08 | 1.54(0.21) |
|  | TT | - | - | -2.29 | 1.51(0.22) |
| Verbal communication | A | +0.15 | 0.74(0.39) | +0.48 | **4.71(0.03)** |
|  | T | -0.15 |  | -0.48 |  |
|  | AA | - | - | +1.64 | **8.56(0.003)** |
|  | AT | - | - | -1.64 | 2.31(0.13) |
|  | TT | - | - | -1.64 | 0.79(0.37) |
| Nonverbal communication | A | +0.17 | 0.74(0.39) | +0.45 | **4.08(0.04)** |
|  | T | -0.17 |  | -0.45 |  |
|  | AA | - | - | +1.35 | **7.25(0.007)** |
|  | AT | - | - | -1.36 | 2.22(0.14) |
|  | TT | - | - | -1.33 | 0.61(0.43) |
| Activity level | A | +0.16 | 0.84(0.36) | +0.71 | **6.74(0.009)** |
|  | T | -0.16 |  | -0.71 |  |
|  | AA | - | - | +1.49 | **8.32(0.003)** |
|  | AT | - | - | -1.40 | 1.30(0.25) |
|  | TT | - | - | -1.74 | 2.20(0.14) |
| Level and consistency of Intellectual Response | A | +0.29 | 2.52(0.11) | +0.52 | **5.12(0.02)** |
|  | T | -0.29 |  | -0.52 |  |
|  | AA | - | - | +1.67 | **7.43(0.006)** |
|  | AT | - | - | -1.62 | 1.08(0.30) |
|  | TT | - | - | -1.79 | 1.55(0.21) |
| General impression | A | +0.19 | 1.16(0.28) | +0.42 | **3.74(0.05)** |
|  | T | -0.19 |  | -0.42 |  |
|  | AA | - | - | +1.47 | **6.41(0.01)** |
|  | AT | - | - | -1.46 | 1.46(0.23) |
|  | TT | - | - | -1.50 | 0.78(0.38) |
